# Supplementary material for: Overeaters Anonymous: An Overlooked Intervention for Binge Eating Disorder
Source: Int J Environ Res Public Health. 2021 Jul 8;18(14):7303. doi: 10.3390/ijerph18147303 (PMC8305393; doi:10.3390/ijerph18147303)
Supplement: Supplementary file 1 [file ijerph-18-07303-s001.zip › ijerph-1234144-supplementary.pdf]

International Journal of
Environmental Research and Public Health

Supplementary Text S1: Excerpts from the Text Alcoholics Anonymous Describing the Twelve-Step Perspective on Spirituality and Spiritual Experiences

Brenna Bray, Boris C. Rodríguez-Martín, David A. Wiss, Christine E. Bray, and Heather Zwickey

Alcoholics Anonymous (AA) began in 1934 when alcoholism was not well understood. The conventional treatments were not efficacious, and the prognosis was poor[1,2]. Carl Jung, a prominent psychoanalyst contemporary who treated alcoholism, noted that some chronic alcoholics had what he called a "vital spiritual experience" that seemed to allow them to recover[3]. Jung described these experiences as "phenomena" in which new ideas, emotions, attitudes, and motives became dominant in the individuals' lives[3]. These insights led one of Jung's patients to join a Christian Evangelical movement of the time (the Oxford Group[4]) founded upon the principle of "giving one's life to God," and carrying the message of salvation to others in need[3-6]. This conversion experience was shared with AA's founder, who expressed hesitation toward the religious emphasis, and was invited to "choose [his] own conception of God[7]," which could be spiritual rather than denominational or religious[3]. Therefore, AA, OA, and other twelve-step programs endorse the importance of non-religious, non-denominational spiritual growth. Below are included two excerpts from the text Alcoholics Anonymous[3] that describe the twelve-step perspective on spirituality and spiritual experiences.

S1.1 Excerpts from Chapter 4, "We Agnostics[8]" in the Book Alcoholics Anonymous[3], the Basic Text for Twelve Step Programs

The following excerpts from Chapter 4 of AA's basic text of the same name (Alcoholics Anonymous[3]) are provided to convey AA (and OA's) views on the role of spirituality in the program:

> *Lack of power, that was our dilemma. We had to find a power by which we could live, and it had to be a Power greater than ourselves. Obviously.…*
>
> *…[Therefore, the] main object [of this book] is to enable you to find a Power greater than yourself which will solve your problem. That means we have written a book which we believe to be spiritual as well as moral. … Much to our relief, we discovered we did not need to consider another's conception of God. Our own conception, however inadequate, was sufficient…*
>
> *…When, therefore, we speak to you of God, we mean your own conception of God. This applies, too, to other spiritual expressions which you find in this book. Do not let any prejudice you may have against spiritual terms deter you from honestly asking yourself what they mean to you[8].*

S1.2 Appendix II: "Spiritual Experience[9]" in the Book Alcoholics Anonymous[3], the Basic Text for Twelve-Step Programs

The following text is provided in Appendix II of AA's basic text of the same name (Alcoholics Anonymous[3]), and describes what is meant by the terms "spiritual experience" and "spiritual awakening" when used in Twelve-Step literature:

> *The terms "spiritual experience" and "spiritual awakening" are used many times in this book which, upon careful reading, shows that the personality change sufficient to bring about recovery from alcoholism has manifested itself among us in many different forms.*
>
> *Yet it is true that our first printing gave many readers the impression that these personality changes, or religious experiences, must be in the nature of sudden and spectacular upheavals. Happily for everyone, this conclusion is erroneous. In the first few chapters a number of sudden revolutionary changes are described. Though it was not our intention to create such*

an impression, many alcoholics have nevertheless concluded that in order to recover they must acquire an immediate and overwhelming "God-consciousness" followed at once by a vast change in feeling and outlook.

Among our rapidly growing membership of thousands of alcoholics such transformations, though frequent, are by no means the rule. Most of our experiences are what the psychologist William James calls the "educational variety" because they develop slowly over a period of time. Quite often friends of the newcomer are aware of the difference long before he is himself. He finally realizes that he has undergone a profound alteration in "his reaction to life; that such a change could hardly have been brought about by himself alone. What often takes place in a few months could seldom have been accomplished by years of self-discipline. With few exceptions our members find that they have tapped an unsuspected inner resource which they presently identify with their own conception of a Power greater than themselves.

Most of us think this awareness of a Power greater than ourselves is the essence of spiritual experience. Our more religious members call it "God-consciousness."

Most emphatically we wish to say that any alcoholic capable of honestly facing his problems in the light of our experience can recover, provided he does not close his mind to all spiritual concepts. He can only be defeated by an attitude of intolerance or belligerent denial.

We find that no one need have difficulty with the spirituality of the program. Willingness, honesty and open mindedness are the essentials of recovery. But these are indispensable.

"There is a principle which is a bar against all information, which is proof against all arguments and which cannot fail to keep a man in everlasting ignorance-that principle is contempt prior to investigation." - HERBERT SPENCER[9]

References

1. Talchekar, A. Timeline: History of Addiction Treatment. Available online: https://www.recovery.org/drug-treatment/history/ (accessed on Dec 16, 2020).
2. O'Malley, A. *The Cure of Alcoholism*; Gummersbach, J (Ed.). Herder: St. Louis, MO, 1913. Available online: http://www.williamwhitepapers.com/pr/1913%20O%27Malley%20The%20Cure%20of%20Alcoholism.pdf (accessed on March 7, 2021).
3. Alcoholics Anonymous. *Alcoholics Anonymous: The story of how many thousands of men and women have recovered from alcoholism*, 4 ed.; Alcoholics Anonymous World Services, Inc.: New York, NY, 2001.
4. The Layman with a Notebook; Grensted, L.A.. *What is the Oxford Group* [microform]; Oxford University Press: New York, 1933. Available online: https://archive.org/details/MN41558ucmf_6/page/n7/mode/2up (accessed on April 22, 2021).
5. Alcoholics Anonymous. *'Pass It On:' The story of Bill Wilson and how the A.A. message reached the world*; Alcoholics Anonymous World Services, Inc.: New York, NY, 1984.
6. Dubiel, R.M. *The Road to Fellowship: The Role of the Emmanuel Movement and the Jacoby Club in the Development of Alcoholics Anonymous*; iUniverse, Inc.: New York, 2004.
7. Alcoholics Anonymous. Bill's Story. In *Alcoholics Anonymous: The story of how many thousands of men and women have recovered from alcoholism*, 4 ed.; Alcoholics Anonymous World Services, Inc.: New York, NY, 2001; p. 54. Available online: aa.org/assets/en_US?en_bigbook_Chapt1.pdf (accessed July 6, 2021).
8. Alcoholics Anonymous. Chapter 4: We Agnostics. In *Alcoholics Anonymous: The story of how many thousands of men and women have recovered from alcoholism*, 4 ed.; Alcoholics Anonymous World Services, Inc.: New York, NY, 2001; pp. 44 - 57. Available online: aa.org/assets/en_US/en_bigbook_chapt4.pdf (accessed July 6, 2021).
9. Alcoholics Anonymous. Appendix II: Spiritual Experience. In *Alcoholics Anonymous: The story of how many thousands of men and women have recovered from alcoholism*, 4 ed.; Alcoholics Anonymous World Services, Inc.: New York, NY, 2001; pp. 567-568. Available online: aa.org/assets/en_US/en_bigbook_appendiceii.pdf (accessed July 6, 2021).
